# Albumin Oxidation Status in Sepsis Patients Treated With Albumin or Crystalloids

**DOI:** 10.3389/fphys.2021.682877

**Published:** 2021-08-06

**Authors:** Matteo Bonifazi, Jennifer Meessen, Alba Pérez, Francesco Vasques, Mattia Busana, Francesco Vassalli, Deborah Novelli, Roberto Bernasconi, Chiara Signori, Serge Masson, Federica Romitti, Lorenzo Giosa, Matteo Macrì, Iacopo Pasticci, Maria Michela Palumbo, Francisco Mota, Montserrat Costa, Pietro Caironi, Roberto Latini, Michael Quintel, Luciano Gattinoni

**Affiliations:** ^1^Department of Anaesthesiology, Emergency and Intensive Care Medicine, University of Goettingen, Göttingen, Germany; ^2^Department of Cardiovascular Medicine, Istituto di Ricerche Farmacologiche Mario Negri IRCCS, Milan, Italy; ^3^Bioscience Research Group, Grifols, Barcelona, Spain; ^4^Medical Affairs, Grifols, Barcelona, Spain; ^5^Department of Anaesthesia and Critical Care, AOU “S. Luigi Gonzaga, Turin, Italy; ^6^Department of Oncology, University of Turin, Turin, Italy

**Keywords:** albumin, oxidation, sepsis, shock, resuscitation, oxidative stress

## Abstract

Inflammation and oxidative stress characterize sepsis and determine its severity. In this study, we investigated the relationship between albumin oxidation and sepsis severity in a selected cohort of patients from the Albumin Italian Outcome Study (ALBIOS). A retrospective analysis was conducted on the oxidation forms of human albumin [human mercapto-albumin (HMA), human non-mercapto-albumin form 1 (HNA1) and human non-mercapto-albumin form 2 (HNA2)] in 60 patients with severe sepsis or septic shock and 21 healthy controls. The sepsis patients were randomized (1:1) to treatment with 20% albumin and crystalloid solution or crystalloid solution alone. The albumin oxidation forms were measured at day 1 and day 7. To assess the albumin oxidation forms as a function of oxidative stress, the 60 sepsis patients, regardless of the treatment, were grouped based on baseline sequential organ failure assessment (SOFA) score as surrogate marker of oxidative stress. At day 1, septic patients had significantly lower levels of HMA and higher levels of HNA1 and HNA2 than healthy controls. HMA and HNA1 concentrations were similar in patients treated with albumin or crystalloids at day 1, while HNA2 concentration was significantly greater in albumin-treated patients (*p* < 0.001). On day 7, HMA was significantly higher in albumin-treated patients, while HNA2 significantly increased only in the crystalloids-treated group, reaching values comparable with the albumin group. When pooling the septic patients regardless of treatment, albumin oxidation was similar across all SOFA groups at day 1, but at day 7 HMA was lower at higher SOFA scores. Mortality rate was independently associated with albumin oxidation levels measured at day 7 (HMA log-rank = 0.027 and HNA2 log-rank = 0.002), irrespective of treatment group. In adjusted regression analyses for 90-day mortality, this effect remained significant for HMA and HNA2. Our data suggest that the oxidation status of albumin is modified according to the time of exposure to oxidative stress (differences between day 1 and day 7). After 7 days of treatment, lower SOFA scores correlate with higher albumin antioxidant capacity. The trend toward a positive effect of albumin treatment, while not statistically significant, warrants further investigation.

## Introduction

Human serum albumin, through its cysteine-associated thiol group (–SH) at position 34, is the main antioxidant compound in the extracellular compartment (Anraku et al., 2013). Albumin continuously circulates from the intravascular compartment to the interstitial space and again into the intravascular compartment through certain capillaries that are permeable to albumin. The half-life of serum albumin is approximately 21 days. During this time, albumin is exposed to the action of free radicals in the extracellular compartment. Therefore, even in normal conditions, albumin may exist in three forms (Colombo et al., 2012; Nakashima et al., 2018):

- *Human mercapto-albumin (HMA)*, in which the thiol groups are present in their reduced form (–SH).
- *Human non-mercapto-albumin 1 (HNA1)*, in which oxidation of the reduced thiol group (–SH) results in a disulfide bond (S-S; this oxidation may be reversible, as the cysteine thiol group may return to its reduced state by regeneration through other antioxidant molecules).
- *Human non-mercapto-albumin 2 (HNA2)*, in which the oxidation of the reduced thiol group (–SH) is irreversible due to binding with sulfonic and sulfinic acid, or to nitrosilation (Colombo et al., 2012) through the reaction of the reduced thiol group (–SH) with nitric oxide.

The extent to which albumin molecules are oxidized by free radicals (reversibly or irreversibly) proportionally decreases its antioxidant capabilities (Matsuyama et al., 2009; Colombo et al., 2012). While there is consistent evidence of the consequences of albumin oxidation in patients with cirrhosis or decompensated hepatic failure (Mera et al., 2005; Arroyo et al., 2014; Das et al., 2017), the literature describing the oxidation status of albumin in sepsis is scant (Quinlan et al., 1998; Colombo et al., 2012). Severe sepsis and septic shock, however, are well-recognized cases of a free radical storm (Huet et al., 2007; Víctor et al., 2009; Galley, 2011; Bavunoglu et al., 2016).

In this study, we investigated the characteristics of albumin oxidation status and the concentrations of several biomarkers in a subset of patients with severe sepsis or septic shock from the Albumin Italian Outcome Study (ALBIOS; Caironi et al., 2014).

## Materials and Methods

### Study Population

The study population consisted of a total of 60 patients, 40 with septic shock and 20 with severe sepsis (Singer et al., 2016; Rhodes et al., 2017; Vasques et al., 2018), selected from participants to the ALBIOS study (Caironi et al., 2014). In this multicenter, open-label trial, we randomly assigned 1,818 patients with severe sepsis and septic shock, in 100 intensive care units (ICUs), to receive either 20% albumin and crystalloid solution or crystalloid solution alone. Patients were randomized (1:1) to treatment with albumin plus crystalloids (referred as albumin-treated group) or treatment with crystalloids alone. Patients were randomly selected for assessment of the albumin redox state from patients with plasma samples available at day 1 and day 7. Selections were balanced for treatment arm and shock. Control samples of plasma (*n* = 21, aged 48–80) from healthy (not diagnosed with Alzheimer Disease) age-matched subjects were kindly supplied by ACE Foundation – Catalan Institute of Applied Neurosciences (Barcelona, Spain) from their sample repository.

### Albumin Oxidation Forms

The oxidative state of albumin was measured at days 1 and 7. To assess the redox state of albumin in plasma samples, anionic exchange HPLC (Waters, Milford, MA) was performed using a Shodex-Asahipak ES-502N column (Showa Denko Europe GmbH, Munich, Germany) coupled to a fluorescent detector (Waters) as previously described (Hayashi et al., 2000; Soejima et al., 2004; Oettl and Marsche, 2010). Thawed samples were filtered [0.22 μm pore (Merck Millipore Ltd., Cork, Ireland)] and immediately placed in the carousel of the HPLC apparatus. All the samples were processed in parallel with the controls to ensure sample stability during analysis. Three albumin fractions were identified according to the oxidation status of the thiol group at the Cys34 albumin residue: HMA, HNA1 and HNA2. Percentage was quantified based on peak heights (dividing the peak height of each form by the sum of the peak heights for all albumin forms and multiplying by 100) using Empower software (Waters).

### Biomarkers

Pentraxin 3 (PTX3), presepsin, and chromogranin A (CgA) were assayed at days 1 and 7, as previously described (Mauri et al., 2010; Caironi et al., 2017) as index of the extent of inflammatory response. In addition, N-terminal prohormone of brain natriuretic peptide (NT-proBNP; Masson et al., 2015) was assayed as a marker of volume load and cardiac injury. Redox status was assessed, to document and support the value of sequential organ failure assessment (SOFA) score as global index of the impact of increased oxidative stress on organ function. The following measures were performed: total antioxidant capacity (TAC), myeloperoxidase (MPO), and Malondialdehyde (MDA).

Total antioxidant capacity concentration was measured using the Antioxidant Assay kit (Cayman Chemical, United States, #709001). The assay is based on the ability of antioxidants in the sample to inhibit oxidation of 2,2ꞌ-azino-di-[3-ethylbenzthiazoline sulfonate] to ABTS^+^ by metmyoglobin. The antioxidants in the sample cause a decrease of the absorbance at 750 nm and those values are compared with that of Trolox, a water soluble tocopherol analog, and is quantified as mM Trolox equivalents. MPO was assayed using a colorimetric enzyme-linked immunosorbent assay (ELISA) according to manufacturer’s instructions (R&D Systems, United States). Lipid peroxidation, expressed as MDA, was determined using commercially available kits (MDA Assay Kit, Sigma-Aldrich, Italy, # MAK085). The method relies on the reaction of MDA, an end product of lipid peroxidation, with thiobarbituric acid producing thiobarbituric acid reactive substance, a pink chromogen, which can be measured spectrophotometrically at 532 nm.

### Endpoints

The first endpoint was the difference in the oxidation status of albumin between crystalloids-treated and albumin-treated patients, at day 1 and 7. The secondary endpoint was the assessment of oxidation status of albumin as a function of oxidative stress. In order to investigate the relationship between oxidative stress and the oxidative status of albumin, baseline SOFA score, a surrogate for oxidative stress intensity, was used to stratify patients into three groups (SOFA scores 1–5, 6–8, and 9–13) irrespective of treatment allocation. Oxidative stress, the primary cause of organ failure in sepsis, should be at its peak at baseline compared to the following days.

### Statistical Methods

Baseline clinical characteristics between ALBIOS-treatment arms were compared by means of Chi^2^, or one-way ANOVA, depending on the nature of the variable. The three albumin forms were expressed as both absolute concentrations (g/L), and fractions of total concentration. Comparisons were made by means of one-way ANOVA. Biomarkers were compared between treatment groups by means of Mann–Whitney test for non-parametric variables. Additionally, the change of albumin concentrations and fractions measured at day 1 and day 7 was assessed by means of paired Student’s *t*-test.

As a surrogate for sepsis severity, patients were divided in three groups by baseline SOFA score (arbitrary cut-off: 1–5, 6–8, and 9–13), patients characteristics between these groups were compared by means of Chi^2^ test, one-way ANOVA, or Kruskal-Wallis, as appropriate. Albumin forms at day 1 and day 7 were correlated with the corresponding SOFA scores by means of Pearson or Spearman rank, depending on the distribution of the data.

To assess the association of albumin oxidation forms and 90-day mortality, concentrations of the three albumin-forms were dichotomized using cut-offs estimated from ROC-curves. These dichotomous variables were then included in Kaplan Meier curves and Log Rank analyses. Finally, Cox proportional hazards models were employed (univariately and adjusted for shock and treatment) to assess the independent association of albumin concentration forms and 90-day mortality. Statistical significance was defined as *p* < 0.05. Data were analyzed using SPSS Software (IBM SPSS version 25, Armonk NY, United States).

## Results

Baseline clinical characteristics and the most relevant physiological variables of the patient population were well-balanced between the two ALBIOS treatment arms (Table 1).

**Table 1:** Baseline clinical characteristics by randomization treatment.

			All *N* = 60	Albumin *N* = 30	Crystalloids *N* = 30	*p*
Age	Year		69.9 ± 13.1	71.3 ± 12.8	68.6 ± 13.6	0.431
Sex	Female		26 (43.3%)	13 (43.3%)	13 (43.3%)	0.999
BMI	Kg/m^2^		27.5 ± 6.4	27.4 ± 5.9	27.6 ± 7.1	0.931
Albumin administration	24 h prior to randomization		0	0	0	-
Reason for admission	Medical		36 (60.0%)	17 (56.7%)	19 (63.3%)	0.116
	Elective		3 (5.0%)	0	3 (10.0%)	
	Emergency		21 (35.0%)	13 (43.3%)	8 (26.7%)	
Pre-existing conditions	Liver disease		0	0	0	-
	COPD		6 (10.0%)	4 (13.3%)	2 (6.7%)	0.389
	Chronic renal failure		0	0	0	-
	Immunodeficiency		6 (10.0%)	4 (13.3%)	2 (6.7%)	0.389
	Congestive/Ischemic heart disease		14 (23.3%)	7 (23.3%)	7 (23.3%)	0.999
SAPS II score			49.0 ± 14.9	50.2 ± 15.8	47.8 ± 14.1	0.538
Heart rate	Bpm		104.9 ± 23.8	97.7 ± 25.1	112.1 ± 20.3	0.018
Mean arterial pressure	mmHg		74.0 ± 14.7	73.0 ± 12.4	74.9 ± 16.9	0.625
	mmHg	After 6 h	77.3 ± 11.6	80.3 ± 11.6	74.3 ± 10.9	0.042
Central venous pressure	mmHg		10.6 ± 4.8	10.7 ± 4.7	10.5 ± 4.8	0.846
PaO_2_ / FiO_2_			204 ± 102	202 ± 87	206 ± 117	0.878
Urine output	ml/h		72.9 ± 65.8	79.4 ± 64.6	66.5 ± 67.5	0.454
Lactate	Mmol/L		3.13 ± 3.27	2.70 ± 2.28	3.59 ± 4.07	0.302
Serum albumin	g/L		25.4 ± 4.3	25.4 ± 4.2	25.4 ± 4.4	0.999
Hemoglobin	g/dl		10.9 ± 1.8	11.1 ± 1.8	10.7 ± 1.8	0.472
Serum creatinine	mg/dl		1.80 ± 1.07	1.94 ± 1.34	1.66 ± 0.73	0.319
White blood cells	10^3^/mm^3^		11.6 ± 7.8	12.2 ± 8.1	11.0 ± 7.5	0.543
Central venous O_2_ saturation	%		70.9 ± 10.6	69.6 ± 11.2	72.0 ± 10.2	0.399
Serum bilirubin	(mg/dl)		1.11 ± 0.86	0.98 ± 0.74	1.24 ± 0.95	0.247
Platelet count	(10^9^/L)		198 ± 107	202 ± 120	193 ± 93	0.732
SOFA score			7.3 ± 2.9	7.1 ± 2.9	7.5 ± 2.9	0.606
Positive blood culture			16 (28.1%)	10 (35.7%)	6 (20.7%)	0.206

Data are presented as means ± SD or N (%). Value of *p* for one-way ANOVA or Chi^2^ between Albumin Italian Outcome Study (ALBIOS) treatment groups. BMI, body mass index; COPD, chronic obstructive pulmonary disease.

### Albumin Oxidation and Treatment Arms

**Figure 1:**
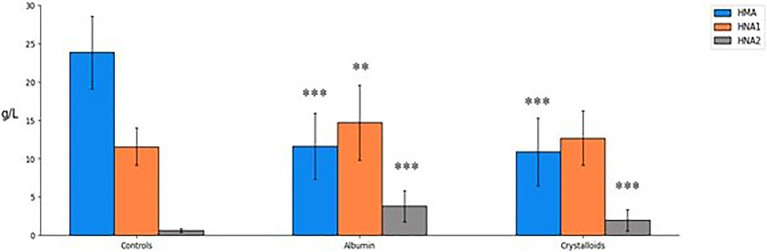
Albumin forms concentration (g/L) in 21 healthy age-matched controls and 60 septic patients on day 1. Mean ± SD, *p* for treated patients, either albumin or crystalloids vs. healthy control groups (one-way ANOVA): **p < 0.01 and ***p < 0.001. HMA, human mercaptoalbumin; HNA1, reversibly oxidized human non-mercaptoalbumin; and HNA2, irreversibly oxidized human non-mercaptoalbumin.

The absolute concentrations of the three forms of albumin (HMA, HNA1, and HNA2) at day 1 are presented in Table 2. Between the two treatment groups, significant differences were found only in HNA2 concentration at day 1. Septic patients treated with albumin had higher concentrations of HNA2 as compared to those treated with crystalloids (3.8 ± 2.0 vs. 2.0 ± 1.4 g/L, *p* < 0.0001). On day 1, HMA concentrations in sepsis patients were significantly reduced in albumin and crystalloids groups compared to healthy controls and to a similar extent. Conversely, the oxidized form HNA2 was significantly increased in both treatment groups compared to healthy controls (Figure 1; Supplementary Table 1).

**Table 2:** Forms of albumin by study treatment on day 1 and 7.

			All *N* = 60	Albumin *N* = 30	Crystalloids *N* = 30	*p*
Concentration g/L	HMA	Day 1	11.2 ± 4.3	11.3 ± 4.3	10.9 ± 4.4	0.562
		Day 7	9.1 ± 4.2	10.2 ± 4.5	7.9 ± 3.7	0.039
	HNA1	Day 1	13.7 ± 4.4	14.7 ± 4.9	12.7 ± 3.5	0.073
		Day 7	15.1 ± 4.0	16.1 ± 4.0	14.0 ± 3.7	0.037
	HNA2	Day 1	2.9 ± 2.0	3.8 ± 2.0	2.0 ± 1.4	3.5 × 10^−4^
		Day 7	3.4 ± 2.4	4.2 ± 2.6	2.6 ± 1.9	0.008
	Total serum albumin	Day 1	27.9 ± 4.7	30.1 ± 4.2	25.7 ± 4.1	1.4 × 10^−4^
		Day 7	27.5 ± 4.7	30.5 ± 2.3	24.5 ± 4.6	3.2 × 10^−8^
Proportion	HMA (%)	Day 1	40.3 ± 14.4	38.8 ± 14.0	41.7 ± 14.8	0.431
		Day 7	32.9 ± 13.9	33.4 ± 14.6	32.3 ± 13.4	0.751
	HNA1 (%)	Day 1	49.6 ± 13.7	48.6 ± 11.9	50.6 ± 15.4	0.566
		Day 7	55.1 ± 12.4	52.7 ± 12.3	57.4 ± 12.3	0.142
	HNA2 (%)	Day 1	10.2 ± 6.4	12.7 ± 7.0	7.7 ± 4.7	0.002
		Day 7	12.1 ± 7.5	13.9 ± 8.3	10.3 ± 6.4	0.067

Data are presented as means ± SD. Value of *p* for one way ANOVA between ALBIOS treatment groups. HMA, human mercaptoalbumin; HNA1, reversibly oxidized human non-mercaptoalbumin; and HNA2, irreversibly oxidized human non-mercaptoalbumin.

### Time Course of Albumin Oxidation

As a direct consequence of treatment, patients infused with albumin had significantly higher total albumin (assayed in local laboratories) at day 1 and day 7 compared to crystalloids-treated patients (*p* < 0.0001). As expected, the difference in albumin concentration between groups was larger at day 7 than at day 1 (Table 2).

**Figure 2:**
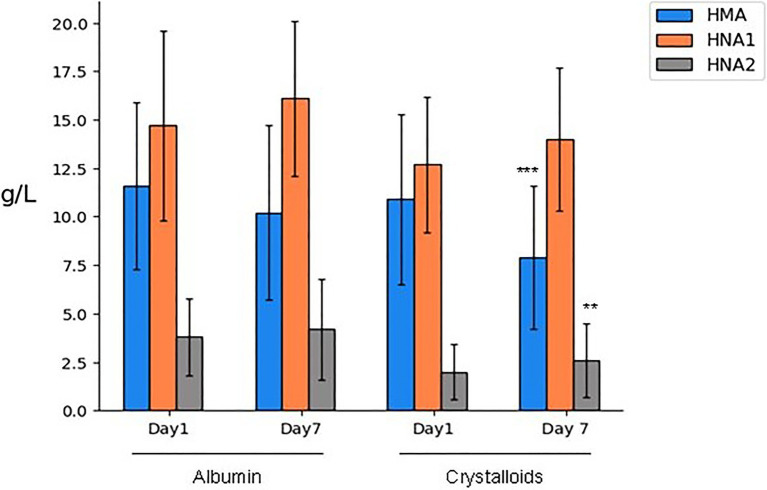
Albumin forms expressed in amounts (g/L) after 1 or 7 days of treatment. Mean ± SD, *p* for one-way ANOVA: **p < 0.01 and ***p < 0.001 day 7 vs. day 1, both for albumin- and crystalloids-treated patients. HMA, human mercaptoalbumin; HNA1, reversibly oxidized human non-mercaptoalbumin; and HNA2, irreversibly oxidized human non-mercaptoalbumin.

Human mercapto-albumin and HNA1 concentrations were similar between patient groups on day 1. However, on day 7, both forms were significantly higher in those patients who received albumin. HNA2 concentration at both time points was higher in the albumin group (Table 2). Comparing the concentrations from day 1 to day 7, only the HMA content in the crystalloids-treated group was significantly decreased (from 10.9 to 7.9 g/L, *p* = 0.007). The HNA2 concentration increased in both groups over time. However, the increase was statistically significant only for the crystalloids-treated group (from 2.0 to 2.6 g/L, *p* = 0.01; Figure 2).

### Proportions of Albumin Forms

In addition to the absolute concentration of the albumin forms, Table 2 presents the percentage of each albumin oxidation form by treatment arm and time point. HNA2 fraction was significantly higher on day 1 but not on day 7 in the albumin-treated group compared to the crystalloids group (Day 1: 12.7 vs. 7.7%, *p* = 0.002; Day 7: 13.9 vs. 10.3%, *p* = 0.067).

### Estimated Oxidative Stress

The association between baseline SOFA score and the “intensity” of sepsis in this population is shown in Table 3. This table presents the values of biomarkers measured at day 1 and day 7. At day 1, most selected biomarkers were significantly higher in the higher SOFA score group. Lactate, frequency of shock and mortality were increased as well. Also MPO and MDA, as markers of oxidative stress, showed a clear positive trend at day 1, increasing at higher SOFA scores. In contrast, by REDOX status as represented by the different albumin forms (both concentration and proportion) were similar at day 1 in agreement with TAC results. At day 7, HNA1 and HNA2 were higher at higher SOFA scores while HMA showed an inverse trend with SOFA groups. TAC results presented the same tendency as HMA indicating therefore an exhaustion of antioxidant capacity over 7 day exposure to sepsis, see Table 3. However, due to low N, the results of these analyses were not statistically significant.

**Table 3:** Circulating biomarkers and albumin oxidative status by baseline sequential organ failure assessment (SOFA) score.

Baseline SOFA score^*^			All *N* = 60*	1–5 *N* = 15	6-8 *N* = 26	9–13 *N* = 17	*p*
SOFA score		baseline	7.3 ± 2.9	3.8 ± 1.4	7.0 ± 0.8	10.9 ± 1.2	-
		Day 1	7.2 ± 3.6	3.6 ± 2.2	6.8 ± 2.3	11.0 ± 2.3	2.6 × 10^−8^
		Day 7	4.5 ± 3.2	2.6 ± 1.4	4.0 ± 2.2	6.8 ± 4.1	3.6 × 10^−4^
PTX3 (ng/ml)		Day 1	57.4 (30.8–181.7)	31.9 (16.1–84.9)	88.5 (35.8–182.3)	77.0 (38.4–213.5)	0.090
		Day 7	19.9 (8.8–34.2)	12.1 (8.5–18.9)	27.5 (9.1–37.1)	22.8 (8.5–53.8)	0.103
Presepsin (ng/L)		Day 1	1,160 (578–2,142)	514 (384–671)	1,160 (593–1,849)	2,898 (1824–4,084)	3.0 × 10^−6^
		Day 7	953 (479–2,010)	474 (307–1,031)	901 (506–1,584)	2016 (779–3,211)	0.003
NT proBNP (ng/L)		Day 1	5,234 (1,930–20,044)	1,097 (514–4,128)	5,343 (2,089–10,484)	19,341 (5,680–35,000)	4.8 × 10^−5^
		Day 7	2050 (494–4,576)	336 (97–1,196)	1872 (517–3,647)	4,314 (2,266–15,974)	1.0 × 10^−4^
CgA (pg/ml)		Day 1	211 (105–400)	99 (67–191)	265 (140–414)	253 (172–432)	0.011
		Day 7	217 (134–505)	107 (80–184)	242 (163–367)	335 (136–827)	0.007
Lactate (mmol/L)		Day 1	2.05 ± 1.51	1.81 ± 1.62	1.60 ± 0.69	3.08 ± 1.98	0.006
		Day 7	1.32 ± 0.51	1.12 ± 0.41	1.28 ± 0.41	1.52 ± 0.68	0.150
Shock			38 (65.5%)	5 (33.3%)	17 (65.4%)	16 (94.1%)	0.001
90 day mortality			20 (34.5%)	2 (13.3%)	9 (34.6%)	9 (52.9%)	0.080
Concentration	HMA (g/L)	Day 1	11.2 ± 4.3	12.6 ± 2.9	11.3 ± 4.5	10.1 ± 4.4	0.224
		Day 7	9.1 ± 4.2	12.2 ± 3.2	8.9 ± 4.3	7.2 ± 3.3	0.002
	HNA1 (g/L)	Day 1	13.7 ± 4.4	14.4 ± 3.6	13.5 ± 5.1	13.5 ± 4.1	0.597
		Day 7	15.1 ± 4.0	14.5 ± 3.8	14.4 ± 4.0	16.5 ± 4.1	0.209
	HNA2 (g/L)	Day 1	2.9 ± 2.0	2.3 ± 1.2	2.6 ± 1.6	3.3 ± 2.1	0.322
		Day 7	3.4 ± 2.4	2.6 ± 1.5	3.2 ± 1.8	3.7 ± 2.2	0.169
Proportion	HMA (%)	Day 1	40.3 ± 14.4	43.4 ± 9.6	41.1 ± 14.7	37.6 ± 15.9	0.514
		Day 7	32.9 ± 13.9	42.1 ± 11.2	33.4 ± 14.1	26.4 ± 10.2	0.003
	HNA1 (%)	Day 1	49.6 ± 13.7	48.8 ± 8.3	49.6 ± 15.6	50.9 ± 15.1	0.926
		Day 7	55.1 ± 12.4	49.1 ± 10.4	55.0 ± 14.2	60.4 ± 9.4	0.028
	HNA2 (%)	Day 1	10.2 ± 6.4	7.7 ± 4.0	9.3 ± 4.9	11.6 ± 6.1	0.126
		Day 7	12.1 ± 7.5	8.8 ± 4.6	11.7 ± 5.9	13.2 ± 6.3	0.113
TAC (mmol/L)		Day 1	3.48 ± 1.06	3.51 ± 1.01	3.52 ± 1.21	3.49 ± 0.94	0.645
		Day 7	3.45 ± 0.97	3.59 ± 0.89	3.51 ± 1.14	3.32 ± 0.79	0.725
MPO (ng/ml)		Day 1	105.0 (56.7–282.3)	72.6 (37.4–130.0)	101.8 (67.6–229.3)	244.7 (116.8–423.8)	0.042
		Day 7	59.6 (39.8–116.0)	50.5 (24.4–97.4)	53.1 (39.4–104.5)	93.5 (42.8–133.5)	0.371
MDA (nmol/L)		Day 1	1.67 ± 2.76	0.98 ± 0.78	1.26 ± 1.37	2.94 ± 4.70	0.082
		Day 7	2.23 ± 3.28	2.81 ± 3.99	1.44 ± 1.68	2.85 ± 4.41	0.289

Data are presented as means ± SD, median [IQR] or N (%).Value of *p* one way ANOVA, Kruskal-Wallis test or Chi^2^ between baseline SOFA score groups. PTX3, pentraxin-3; NTproBNP, NT-pro Brain Naturetic Peptide; CgA, chromogranin A; HMA, human mercaptoalbumin; HNA1, reversibly oxidized human non-mercaptoalbumin; HNA2, irreversibly oxidized human non-mercaptoalbumin; TAC, total antioxidant activity; MPO, myeloperoxidase; and MDA, malondialdehyde.

* Missing SOFA score at baseline for 2 persons.

### Time Course of Oxidative Stress

At day 7, the SOFA score decreased across the SOFA groups. Expectedly, the SOFA score at day 7 was higher in those who started with a higher SOFA score at baseline (*p* < 0.0001). Most of the biomarkers, except CgA, decreased from day 1 to day 7, suggesting a decrease in the extent of inflammatory activation and associated oxidative stress in these patients over time. However, the patients in the higher SOFA score group had significantly higher levels of most biomarkers.

**Figure 3:**
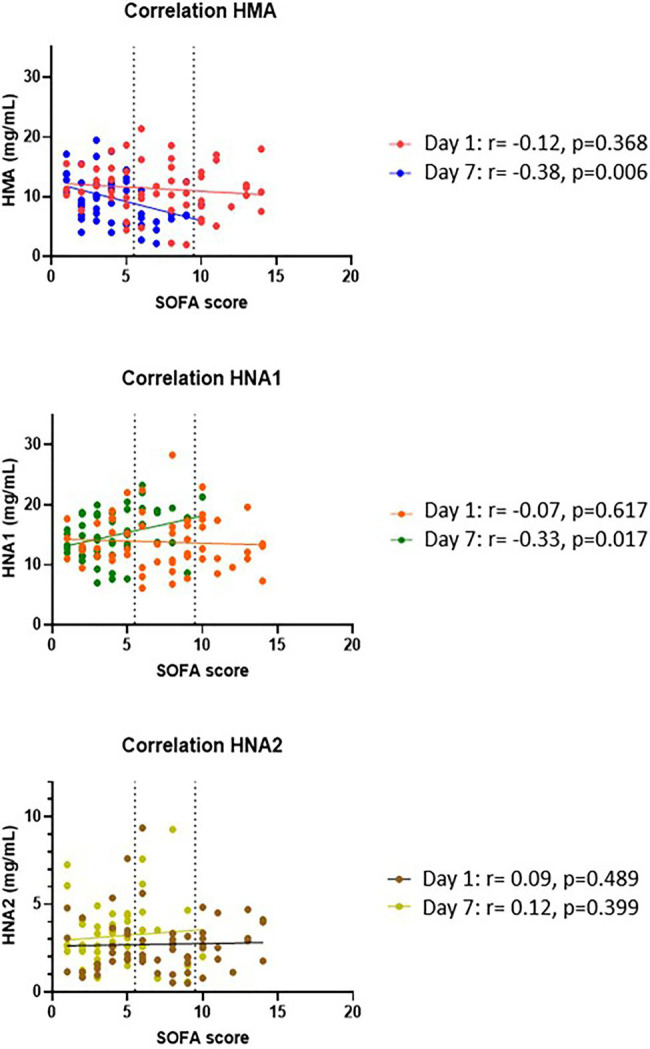
Correlation of albumin oxidation forms and SOFA score. Albumin forms at day 1 and day 7were correlated with the corresponding SOFA scores by means of Pearson or Spearmen rank, depending on the distribution of the data.

Regarding the oxidation status, the decrease of HMA from day 1 to day 7, was larger for patients in the higher SOFA-group as compared to low-SOFA (−0.4 g/L in low SOFA and −2.9 in high SOFA), while HNA1-concentration had a larger increase in high SOFA-group (+0.1 vs. +3.0 g/L). Unexpectedly, HNA2 concentration increase did not differ substantially among baseline SOFA groups, as shown in Table 3. For proportions of the albumin-forms, differences were only found at day 7 with the lowest-SOFA-group having higher percentages of HMA and lower percentages of HNA1. This was further confirmed in a correlation analysis shown in Figure 3. The same trend was visible when limiting the analysis to septic shock patients only, see Supplementary Table 2. When focusing on the 40 patients with septic shock, the trend was even more marked, though it did not reach statistical significance.

### Mortality

The relationship of albumin oxidation forms and mortality was explored by means of ROC curves. Using these curves, the optimal cut-offs for discriminating between surviving and deceased patients were identified: 8.87, 14.99, and 2.32 g/L for HMA, HNA1 and HNA2, respectively. Upon creating binary variables including this cut-off and inserting these in Kaplan–Meier curves (Table 4), both HMA and HNA2 showed significant effects (*p* = 0.040 and *p* = 0.006).

**Table 4:** Regression analyses for day-7 albumin forms and 90-day mortality.

	ROC analysis			Optimal cut-off	Kaplan-Meier	Univariate Cox regression			Adjusted Cox regression^*^		
	AUC	95% CI	*p*		Log rank *p*	OR	95% CI	*p*	OR	95% CI	*p*
HMA	0.66	0.51–0.81	0.040	8.87 g/L	0.011	3.16	1.24–8.09	0.016	3.90	1.31–11.62	0.014
HNA1	0.55	0.39–0.70	0.562	14.99 g/L	0.184	0.57	0.24–1.33	0.191	0.63	0.26–1.55	0.313
HNA2	0.71	0.58–0.85	0.006	2.32 g/L	0.005	0.16	0.04–0.71	0.015	0.16	0.04–0.72	0.017

Oxidation status was dichotomized based on the optimal cut-off as identified in ROC-analyses. These binary variables were then included in Kaplan-Meier curves as well as Cox proportional hazard models. Reference category for regression analyses is the highest group. HMA, human mercaptoalbumin; HNA1, reversibly oxidized human non-mercaptoalbumin; and HNA2, irreversibly oxidized human non-mercaptoalbumin.

* The adjusted models were adjusted for treatment and shock.

We then included these binary variables in Cox proportional hazard analyses: univariate, lower HMA and higher HNA2 concentrations at day 7 were associated with a significant increase in 90-day mortality [HMA: OR = 3.2 (95% CI: 1.2–8.1), *p* = 0.016; HNA2: OR = 0.2 (0.0–0.7), *p* = 0.015]. After adjusting for treatment and shock in the regression analyses, these results remained significant: [HMA: OR=3.9 (95% CI: 1.3–11.6), *p* = 0.014; HNA2: OR = 0.2 (0.0–0.7), *p* = 0.017].

## Discussion

In this study, we found: (1) albumin oxidation was far higher in septic patients than in normal subjects; (2) treatment with albumin and crystalloids appeared to be able to prevent the decrease in HMA observed after 7 days of treatment with crystalloids alone; (3) time and level of exposure to oxidative stress, as inferred from the baseline SOFA score, played a substantial role in determining the oxidation status of albumin and (4) in the change of circulating biomarkers; and (5) HMA and HNA2 are in opposing fashion associated with 90-day mortality independent of shock or treatment.

It is well-known that the development of oxidative stress is a distinctive feature of sepsis (Salvemini and Cuzzocrea, 2002). Oxidative stress is defined as the condition in which reactive oxidative species (ROS; free oxygen radicals; Nagar et al., 2018) and other free radicals, such as reactive nitrogen species (RNS; Park and Zmijewski, 2017) sharply increase, challenging the antioxidant defenses. In this condition, ROS originating from mitochondria and from the membrane-bound NADPH oxidase facing the extracellular membrane may damage membranes, cytoplasm and intra-nuclear components. Some ROS, however, may exert their action on extracellular molecules, including albumin. This protein may reduce ROS through the oxidation of its thiol-cysteine link group, where three oxidation states of albumin are possible: HMA, HNA1, and HNA2. The redox status of total proteins or the role of other immune proteins were considered marginal in comparison to albumin. Indeed albumin is the most abundant protein in the human body, with an intravascular (blood plasma) concentration range of 35–52 mg/ml, accounting for 50–60% of total plasma proteins in healthy humans. Beyond its role as volume expander and transporter, albumin is the main extracellular antioxidant. In addition, albumin concentration is higher than other antioxidant molecules; therefore, albumin would become the main protein in terms of quantities, with antioxidant capacity.

Not surprisingly, during sepsis a ROS storm may quantitatively alter the oxidation status of albumin leading to a decrease in HMA and an increase in HNA1 and HNA2. In this study, we found that after the 1st day of treatment, the fraction of reduced albumin, HMA, and the fraction of reversibly oxidized albumin, HNA1, were similar between patients treated with albumin or crystalloids. Patients infused with albumin had a significantly higher total albumin on day 1 and showed a significant greater concentration of irreversibly oxidized albumin, HNA2. The different concentration of HNA2 could be attributed either to natural processes or a greater amount of irreversibly oxidized albumin fraction in the infused albumin. Considering the similar content of HMA, HNA1 in albumin and crystalloids treated patients at day 1, it is tempting to say that the difference of HNA2 was due mainly to continuous oxidative stress in the crystalloid arm and to both oxidative stress and to exogenous infused oxidized albumin in the albumin-treated group (Bar-Or et al., 2005).

Despite the observation that modified albumin (i.e., oxidized albumin) can be internalized and degraded by cells (Arroyo et al., 2014), the half-life of albumin, is about 21 days (Vincent et al., 2014). This indicates that most of the molecules of albumin exposed to the ROS storm in the 7-day interval were present throughout the entire interval. When analyzing changes in albumin oxidation status from day 1 to day 7, only the crystalloids treated patients show a significant decrease in HMA content on top of a significant increase in HNA2. Altogether, these results suggest that patients treated with albumin would show a better evolution of the albumin oxidation profile from day 1 to day 7 than crystalloids-treated patients.

Moreover, we hypothesized that the baseline SOFA score could be used as a surrogate marker for the ROS storm, as the blow phase of sepsis and septic shock is highest in the initial period of the syndrome and declines a few days later (Kumar et al., 2018). Dividing the population into groups by baseline SOFA (Barle et al., 2002; Mantzarlis et al., 2017; Kumar et al., 2018) independent of the treatment, we found that most biomarkers were significantly increased from lowest to highest SOFA groups and these markers declined over the following week. This suggested an improvement over time in the conditions of patients that survived to day 7.

This supports the hypothesis that the baseline SOFA score was proportional to the intensity of the ROS storm and that this intensity decreased or vanished over 1 week. In support of the assumption that baseline SOFA score reflects intensity of oxidative stress is the higher MDA and MPO concentrations at higher SOFA scores (Table 3). Altogether these data support the close relation between severity of sepsis, intensity of oxidative stress, and organ injury (e.g., SOFA score).

While a positive correlation between biomarker levels and SOFA score may exist, this relationship does not seem to occur between albumin oxidation forms and sepsis severity scores when analyzing pooled patients.

Unexpectedly, albumin oxidation forms were similar at day 1 throughout the SOFA groups. At first, this finding seemed paradoxical. Indeed, when ROS intensity peaked at day 1, the oxidation of albumin was independent of ROS intensity. However, when the ROS storm had decreased by day 7, oxidation status was sharply different between patients exposed to higher or lower intensity of ROS storm. In particular, HMA became remarkably different at day 7 indicating an ongoing consumption of the antioxidant body stores after 1 week. This would be more important for patients with higher baseline SOFA scores. In agreement with this observation, a weak correlation between higher HMA values and lower SOFA scores at day 7 was observed. Similarly, TAC as a measure of the total antioxidant capacity, while similar across SOFA scores at day 1, tended to be higher at lower SOFA scores at day 7.

It is possible that the mechanisms responsible for returning oxidized albumin to its initial reduced status are fully operational at the onset of the syndrome and decrease with time. Furthermore, when separating albumin and crystalloids treatment arms among SOFA groups, after 7 days of treatment, higher percentages of albumin-treated patients (80.8 vs. 63.0%, *p* = 0.332) were found in the group with lowest SOFA scores, Supplementary Table 3. This trend in favor of albumin supplementation may well be due to a higher prevalence of low SOFA score at baseline, 35.7% in albumin vs. 16.7% in crystalloids. Although the differences are not statistically significant, this aspect warrants further investigation on a larger number of patients.

Considering all these data, we propose the following model: ROS originating from both the mitochondria and the membrane-bound NADPH oxidase reach the extracellular matrix, where they randomly oxidize a fraction of circulating albumin. If this occurs, the decrease in the fraction of reduced albumin and the increase in the fraction of oxidized albumin would correlate with the intensity of the ROS storm. On day 1, when antioxidant mechanisms are fully functional, the fractions of albumin are similar suggesting preservation of the capability of the body to reduce albumin. With time, although ROS most likely decline oxidized albumin progressively increases. Due to the long half-life of albumin, the same molecules are repeatedly exposed to oxidative stress. Therefore, the level of oxidized albumin is an indicator of the cumulative oxidative stress that the molecules undergo during the exposure period. Not surprisingly, the mortality rate was found to be associated with the oxidation level of albumin on day 7, i.e., with the cumulative intensity of oxidative stress. Peak capacity of albumin oxidation was not reached according to the absolute values of HNA2. Indeed it has been reported that in other pathologies with strong inflammatory and oxidative profiles such as acute on chronic liver failure, a condition with many similarities with sepsis, endogenous albumin can reach higher HNA2 absolute values (up to 30%) than those detected in our work (Clària et al., 2016).

While the oxidative fractions of albumin are related to the intensity of stress, the absolute concentrations of each fraction in g/L indicate how much albumin was available to react with ROS and how much of albumin already underwent this reaction, reversibly or irreversibly. Since ROS can induce chain reactions, the oxidation of albumin indicates that other molecules are substantially spared from the oxidative cascade. In previous studies (Bourdon and Blache, 2001; Lang et al., 2004; Liu et al., 2012; Vincent et al., 2016), we found an indication of the benefits of albumin administration in septic shock patients, where the intensity of oxidative stress was higher. It is possible that the sparing action of albumin, which is proportional to its concentration, may improve outcome in a fraction of septic shock patients, but this finding needs confirmation.

In conclusion, the data presented here suggest that the oxidation status of albumin in patients with sepsis is related to the time of exposure to oxidative stress (differences between day 1 and day 7). After 7 days of treatment, lower SOFA scores correlate with higher albumin antioxidant capacity. The trend toward a positive effect of albumin treatment, while not statistically significant, warrants further investigation.

## Data Availability Statement

Data is available upon request, to be examined and accepted by the Steering Committee of ALBIOS trial.

## Ethics Statement

This study was reviewed and approved by each Ethics Committee of the 100 centers included in the ALBIOS trial. The patients/participants provided their written informed consent to participate in this study.

## Author Contributions

MBo, JM, RL, and LGa have made substantial contributions to the conception and design of the work. AP, FVq, MBu, FVs, DN, RB, CS, FR, LGi, MM, and IP contributed to the acquisition of data. MBo, JM, AP, SM, RL, and LGa contributed to the analysis and interpretation of data. MBo, JM, MP, FM, MC, PC, RL, MQ, and LGa have drafted the work or substantively revised it. All authors have approved the submitted version (and any substantially modified version that involves the author’s contribution to the study), and have agreed both to be personally accountable for the author’s own contributions and to ensure that questions related to the accuracy or integrity of any part of the work, even ones in which the author was not personally involved, are appropriately investigated, resolved, and the resolution documented in the literature.

## Conflict of Interest

MC, FM, and AP are full-time employees of Grifols, a manufacturer of plasma derivatives. Grifols had no access to clinical data from ALBIOS and did not perform statistical analyses of data, but only provided editorial support. Publication costs of this manuscript were sustained by Grifols. LG and PC received honoraria from Grifols.

## Publisher’s Note

All claims expressed in this article are solely those of the authors and do not necessarily represent those of their affiliated organizations, or those of the publisher, the editors and the reviewers. Any product that may be evaluated in this article, or claim that may be made by its manufacturer, is not guaranteed or endorsed by the publisher.
