# Supplementary material for: Albumin Oxidation Status in Sepsis Patients Treated With Albumin or Crystalloids
Source: Front Physiol. 2021 Aug 6;12:682877. doi: 10.3389/fphys.2021.682877 (PMC8383812; doi:10.3389/fphys.2021.682877)
Supplement: Supplementary file 1 [file Data_Sheet_1.docx]

Supplementary material

Albumin oxidation status in sepsis patients treated with albumin or crystalloids

Matteo Bonifazi MD^1^, Jennifer Meessen PhD^2^, Alba Pérez PhD^3^, Francesco Vasques MD^1^ , Mattia Busana MD^1^ , Francesco Vassalli MD^1^ , Deborah Novelli PhD^2^ , Roberto Bernasconi ^2^, Chiara Signori BSc^2^, Serge Masson PhD^2,4^ , Federica Romitti MD^1^ , Lorenzo Giosa MD^1^, Matteo Macrì MD^1^ , Iacopo Pasticci MD^1^, Maria Palumbo MD^1^ , Francisco Mota Pharm D, MSc^5^ , Montserrat Costa PhD^3^ , Pietro Caironi MD^6^ , Roberto Latini MD^2^ , Michael Quintel MD^1^; Luciano Gattinoni MD*^1^

1. Department of Anaesthesiology, Emergency, and Intensive Care Medicine, University of Goettingen, Göttingen, Germany.

2. Department of Cardiovascular Medicine, Istituto di Ricerche Farmacologiche Mario Negri IRCCS, Milan, Italy.

3. Bioscience Research Group, Grifols, Barcelona, Spain.

4 Present address: Roche Diagnostics, Rotkreuz, Switzerland.

5. Medical Affairs, Grifols, Barcelona, Spain.

6. Department of Anaesthesia and Critical Care, AOU "S. Luigi Gonzaga", Department of Oncology, University of Turin, Turin, Italy.

## **Supplementary Table 1 -** Fractions of albumin oxidation status in 21 healthy volunteers and ALBIOS sepsis patients.

|  | | **Albumin**  **N=30**  **Day 1 of ALBIOS study** | **Crystalloids**  **N=30**  **Day 1 of ALBIOS study** | **Healthy age-matched controls**  **N=21** |
| --- | --- | --- | --- | --- |
| HMA | % | 38.8 ± 14.0 | 41.7 ± 14.8 | 66.1 ± 4.2 |
|  | g/L | 11.3 ± 4.3 | 10.9 ± 4.4 | 23.8 ± 4.7 |
| HNA1 | % | 48.6 ± 11.9 | 50.6 ± 15.4 | 32.2 ± 4.3 |
|  | g/L | 14.7 ± 4.9 | 12.7 ± 3.5 | 11.6 ± 2.4 |
| HNA 2 | % | 12.7 ± 7.0 | 7.7 ± 4.7 | 1.76 ± 0.52 |
|  | g/L | 3.8 ± 2.0 | 2.0 ± 1.4 | 0.64 ± 0.24 |
| Serum albumin | g/L | 30.1 ± 4.2 | 25.7 ± 4.1 | 36.0 ± 6.5 |
| **HMA** - human mercaptoalbumin; **HNA1** - reversibly oxidized human non-mercaptoalbumin; **HNA2** - irreversibly oxidized human non-mercaptoalbumin. | | | | |

**Supplementary Table 2** – Forms of albumin by baseline SOFA score in 40 patients with septic shock.

| **Baseline SOFA Score***  **Shock patients only** | | | **All**  **N=40*** | **1-5**  **N=5** | **6-8**  **N=17** | **9-13**  **N=16** | **P** |
| --- | --- | --- | --- | --- | --- | --- | --- |
| Concentration | HMA  (g/L) | Day 1 | 10.9 ± 4.7 | 12.3 ± 2.8 | 11.5 ± 4.9 | 10.1 ± 4.6 | 0.547 |
|  |  | Day 7 | 8.0 ± 3.9 | 10.6 ± 3.3 | 8.3 ± 4.3 | 7.5 ± 3.2 | 0.253 |
|  | HNA1  (g/L) | Day 1 | 13.0 ± 4.0 | 13.7 ± 3.7 | 12.3 ± 4.1 | 13.6 ± 4.2 | 0.634 |
|  |  | Day 7 | 15.5 ± 4.1 | 15.8 ± 3.3 | 14.2 ± 4.4 | 16.8 ± 4.1 | 0.188 |
|  | HNA2  (g/L) | Day 1 | 3.2 ± 2.2 | 2.6 ± 1.0 | 2.9 ± 1.9 | 3.2 ± 2.1 | 0.845 |
|  |  | Day 7 | 3.9 ± 2.7 | 3.2 ± 1.5 | 3.5 ± 2.0 | 3.7 ± 2.2 | 0.824 |
| Proportion | HMA  (%) | Day 1 | 39.8 ± 15.6 | 43.3 ± 10.3 | 42.4 ± 15.2 | 37.5 ± 16.4 | 0.649 |
|  |  | Day 7 | 29.7 ± 13.7 | 36.2 ± 12.2 | 32.3 ± 15.8 | 27.2 ± 10.1 | 0.280 |
|  | HNA1  (%) | Day 1 | 48.7 ± 14.7 | 47.7 ± 8.4 | 47.4 ± 16.1 | 51.1 ± 15.6 | 0.727 |
|  |  | Day 7 | 56.7 ± 12.6 | 53.1 ± 9.0 | 54.9 ± 15.9 | 60.0 ± 9.6 | 0.354 |
|  | HNA2  (%) | Day 1 | 11.4 ± 7.2 | 9.0 ± 2.7 | 10.2 ± 5.7 | 11.4 ± 6.3 | 0.761 |
|  |  | Day 7 | 13.6 ± 8.3 | 10.7 ± 4.7 | 12.9 ± 6.7 | 12.8 ± 6.3 | 0.943 |
| * baseline SOFA score missing for 2 patients. P-value for one way ANOVA across groups. **HMA** - human mercaptoalbumin; **HNA1** - reversibly oxidized human non-mercaptoalbumin; **HNA2** - irreversibly oxidized human non-mercaptoalbumin. | | | | | | | |

.

.

.

## **Supplementary table 3 –** Treatment by SOFA score groups.

| Total population (N=60) | | **total** | **SOFA score**  **1-5** | **SOFA score**  **6-8** | **SOFA score**  **9-13** | **P** |
| --- | --- | --- | --- | --- | --- | --- |
| Treatment | Albumin baseline | 28 | 10 (35.7%) | 10 (35.7%) | 8 (28.6%) | 0.218 |
|  | Crystalloids baseline | 30 | 5 (16.7%) | 16 (53.3%) | 9 (30.0%) |  |
|  | Albumin day1 | 28 | 11 (39.3%) | 7 (25.0%) | 10 (35.7%) | 0.893 |
|  | Crystalloids day1 | 30 | 10 (33.3%) | 8 (26.7%) | 12 (40.0%) |  |
|  | Albumin day 7 | 25 | 21 (80.8%) | 3 (11.5%) | 2 (7.7%) | 0.332 |
|  | Crystalloids day 7 | 26 | 17 (63.0%) | 7 (25.9%) | 2 (11.1%) |  |
| Shock only (N=40) | | **total** | **SOFA score**  **1-5** | **SOFA score**  **6-8** | **SOFA score**  **9-13** | **P** |
| Treatment | Albumin baseline | 18 | 4 (22.2%) | 6 (33.3%) | 8 (44.4%) | 0.205 |
|  | Crystalloids baseline | 20 | 1 (5.0%) | 11 (55.0%) | 8 (40.0%) |  |
|  | Albumin day1 | 18 | 5 (27.8%) | 5 (27.8%) | 8 (44.4%) | 0.819 |
|  | Crystalloids day1 | 20 | 4 (20.0%) | 7 (35.0%) | 9 (45.0%) |  |
|  | Albumin day 7 | 17 | 14 (82.4%) | 2 (11.8%) | 1 (5.9%) | 0.197 |
|  | Crystalloids day 7 | 20 | 11 (55.0%) | 7 (35.0%) | 2 (10.0%) |  |
| Distribution of patients amongst the SOFA scores at three time points (baseline, day 1 and day 7) by treatment. Percentage is calculated for the distribution within treatment group (row). P for Chi^2^ test. | | | | | | |
